# Incidental discovery and surgical removal of inferior vena cava filter fragment embolization to the right ventricle with pericardial perforation during coronary angiography: a case report

**DOI:** 10.1093/ehjcr/ytaf657

**Published:** 2025-12-20

**Authors:** Benjamin M Easow, Tijin Mathew, Lydia George, Darius G Aliabadi

**Affiliations:** Internal Medicine Residency Program, Southeast Health, 1108 Ross Clark Circle, Dothan, AL 36301, USA; Internal Medicine Residency Program, Southeast Health, 1108 Ross Clark Circle, Dothan, AL 36301, USA; Internal Medicine Residency Program, Southeast Health, 1108 Ross Clark Circle, Dothan, AL 36301, USA; Interventional Cardiologist, Department of Cardiology, Southeast Health, 1108 Ross Clark Circle, Dothan, AL 36301, USA

**Keywords:** Inferior vena cava filter, Filter embolization, Coronary angiography, Pericardial perforation, Coronary artery bypass grafting, Case report

## Abstract

**Background:**

Retrievable inferior vena cava (IVC) filters, when not removed after the period of thromboembolic risk has resolved, are prone to delayed complications such as limb fracture, migration, and embolization. Fragment embolization to the heart can result in pericardial penetration or other life-threatening sequelae yet may remain entirely asymptomatic and identified only incidentally.

**Case summary:**

A 54-year-old woman with a retrievable IVC filter placed in 2009 for pulmonary embolism, during a period of high bleeding risk from active breast cancer treatment, underwent elective left heart catheterization in 2025 for a 2-month history of chest discomfort. Coronary computed tomography (CT) angiography revealed multi-vessel coronary artery disease but did not visualize the migrated filter strut. During angiography, a linear radiopaque foreign body was seen within the right ventricle. A kidney–ureters–bladder radiograph confirmed filter fracture with a missing limb. Echocardiography 2 months earlier showed preserved bi-ventricular function and no pericardial effusion. During coronary artery bypass grafting, a fractured metallic filter limb ∼1–2 cm in length penetrating the pericardium was surgically removed. The remaining filter was left *in situ* with planned radiographic surveillance. Recovery was uneventful.

**Discussion:**

Thin metallic struts may evade CT detection due to in-plane alignment or artefact, and echocardiography may fail to identify fragments lacking haemodynamic significance. Management options include endovascular retrieval, surgical extraction, or observation. This case highlights the importance of structured IVC filter follow-up and timely retrieval to prevent silent but potentially life-threatening complications.

Learning points

- Delayed inferior vena cava (IVC) filter fracture and intra-cardiac migration may remain completely asymptomatic and can be discovered only during unrelated procedures.
- Thin metallic filter fragments may evade detection on computed tomography and echocardiography due to their small size, alignment, and artefact-related limitations.
- Surgical extraction is favoured when a migrated fragment causes perforation or carries risk of arrhythmia or embolization, particularly when percutaneous retrieval is not feasible.
- Coronary angiography can reveal clinically important incidental extra-coronary findings; meticulous review of all fluoroscopic fields is essential.
- Retrievable IVC filters, especially older-generation models, carry a higher risk of fragmentation and embolization—necessitating proactive follow-up and retrieval programs.

## Introduction

Inferior vena cava (IVC) filters are used in patients with contraindications to anticoagulation or recurrent venous thromboembolism. Although intended for temporary use, retrievable filters are frequently left *in situ*, predisposing patients to complications including fracture, migration, penetration, and embolization.^1,2^ Migrated struts may cause arrhythmias, cardiac perforation, tamponade, or sudden death.^3^

Despite known risks, retrieval rates remain low due to fragmented follow-up systems and lack of structured recall programmes.^4^ Many filter-related complications are discovered incidentally on imaging performed for unrelated conditions.

This report describes a patient with a suspected C.R.Bard (BARD) G2 filter placed in 2009 and never retrieved, whose fractured limb embolized to the right ventricle with pericardial penetration and was discovered during coronary angiography in 2025.

## Summary figure

**Figure:**
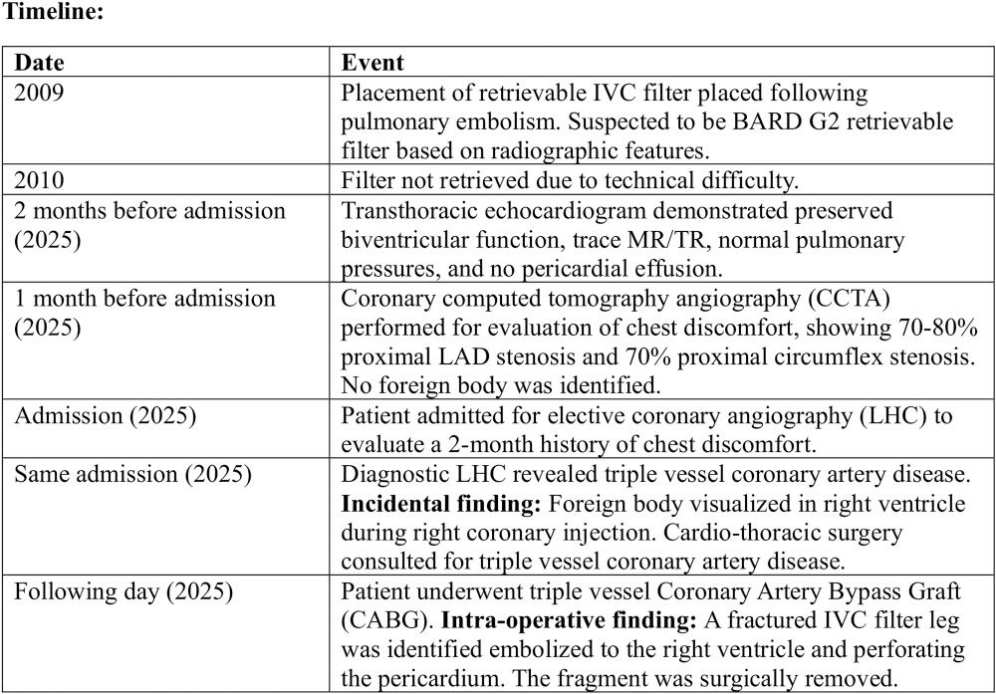


## Case presentation

A 54-year-old woman with hypertension, hyperlipidaemia, chronic pericarditis, a history of breast cancer, and chronic migraines presented with a 2-month history of chest discomfort. She had sustained a pulmonary embolism in 2009 during active cancer treatment, at which time a retrievable IVC filter was placed because anticoagulation was considered high-risk. A retrieval attempt was later aborted due to procedural difficulty.

One month prior to admission, coronary computed tomography angiography (CCTA) demonstrated multi-vessel coronary artery disease, including 70%–80% proximal left anterior descending (LAD) stenosis with a diminutive distal LAD and 70% proximal circumflex stenosis. No intra-cardiac metallic foreign body was identified, likely due to the strut’s small diameter, in-plane alignment, partial-volume averaging, and beam-hardening artefact. A *trans*-thoracic echocardiogram performed 2 months earlier showed normal left and right ventricular size and function with an ejection fraction of 56%, trace mitral and tricuspid regurgitation, an estimated pulmonary artery systolic pressure of 31 mmHg, and no pericardial effusion.

**Figure 1:**
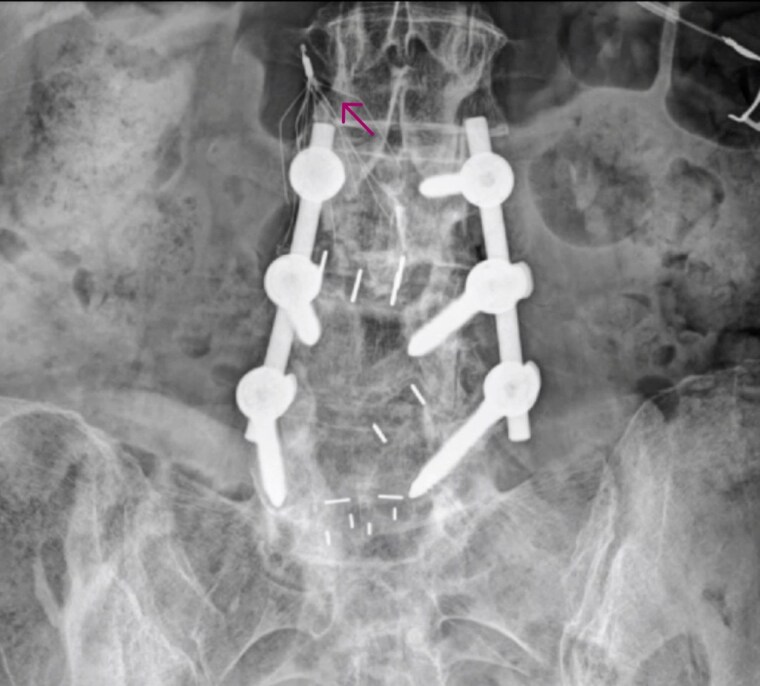
Coronary angiography (right coronary artery injection). Fluoroscopic image demonstrating a linear radiopaque foreign body within the right ventricle (white arrows). The fragment shows partial mobility synchronized with the cardiac cycle. This image corresponds to Supplementary material online, *Video S1*.

She was admitted in 2025 for elective left heart catheterization to further evaluate persistent chest discomfort. Catheterization confirmed severe triple-vessel coronary artery disease, including 50% mid-LAD stenosis tapering to ≥90% distally, 90% proximal obtuse marginal stenosis, and 90% proximal posterior descending artery stenosis, with a widely patent left internal mammary artery. During right coronary artery injection, an unexpected linear radiopaque foreign body was visualized within the right ventricle, partially fixed and partially mobile, moving synchronously with the cardiac cycle (*Figure 1*, Supplementary material online, *Video S1*).

**Figure 2:**
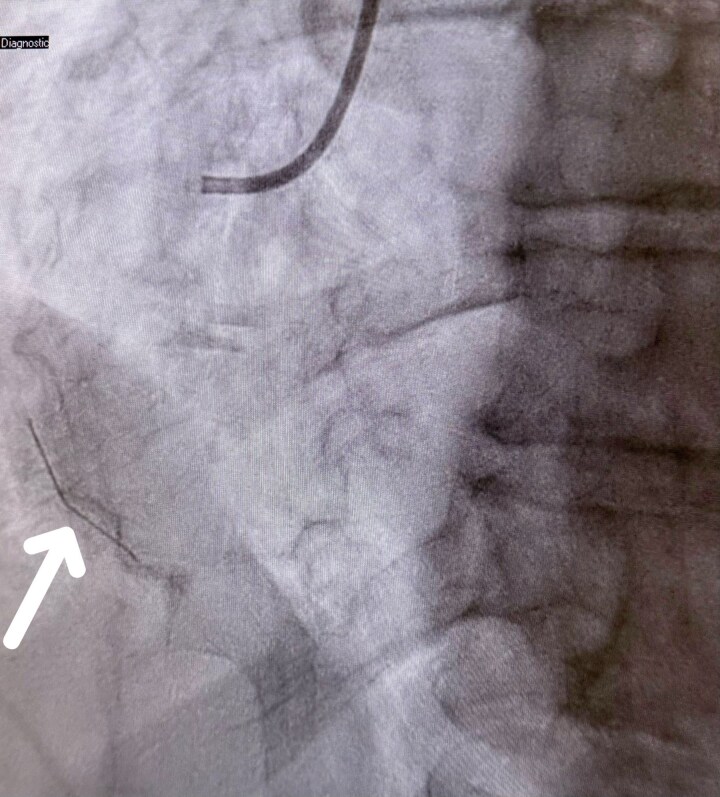
Abdominal radiograph (kidney–ureters–bladder view). Plain abdominal radiograph showing fractured and displaced components of the inferior vena cava filter (arrow), with absence of one limb. Radiographic configuration is most consistent with a retrievable BARD G2 filter design.

A kidneys–ureters–bladder (KUB) abdominal radiograph confirmed IVC filter fragmentation with a missing limb, and the residual configuration was radiographically consistent with a BARD G2 retrievable filter (*Figure 2*). Cardio-thoracic surgery was consulted, and she underwent triple-vessel coronary artery bypass grafting (left internal mammary artery to left anterior descending, and saphenous vein grafts to the obtuse marginal and posterior descending arteries). Intra-operatively, a fractured metallic filter limb measuring ∼1–2 cm was identified within the right ventricle with penetration through the pericardium and was surgically removed without complication.

The remainder of the filter was left *in situ*. Post-operative evaluation for retrieval was deferred, and interval radiographic surveillance was arranged to monitor for further fractures or migration. Her recovery was uneventful, and she was discharged in stable condition on aspirin, clopidogrel, statin therapy, and metoprolol, with close follow-up arranged.

## Discussion

Inferior vena cava filter fracture and migration are well-recognized delayed complications, particularly among older-generation retrievable devices such as the BARD G2 filter. Multiple mechanisms contribute to filter limb fracture, including chronic mechanical fatigue from constant caval motion, prolonged dwell time leading to metal fatigue, asymmetric filter tilt producing uneven loading, endothelial overgrowth that tethers or distorts struts, and long-term caval wall stress.^1–3^ These bio-mechanical forces predispose to progressive weakening and eventual fragmentation, especially in filters left *in situ* for many years. Once detached, embolized fragments may migrate to the heart and cause arrhythmias, perforation, tamponade, or sudden collapse, although as in this case the patient may remain entirely asymptomatic.

Although CCTA has the capability to detect embolized filter fragments, multiple imaging limitations can obscure thin metallic struts. Prior case reports have demonstrated that embolized filter fragments can be identified on computed tomography (CT), including through advanced 3D volume-rendered reconstructions.^5^ However, fine metallic struts may still evade detection when aligned parallel to the imaging plane, when measuring below slice thickness, or when partially obscured by partial-volume averaging, streak artefact, or motion within the right ventricle. Similarly, *trans*-thoracic echocardiography may fail to visualize slender metallic fragments because of limited acoustic reflectivity, shadowing, or the absence of secondary findings such as pericardial effusion. Accordingly, a normal CT or echocardiogram does not exclude the presence of an intra-cardiac metallic foreign body, as demonstrated in this patient.

Once embolization is suspected, initial evaluation typically begins with a KUB radiograph to assess filter integrity. Fluoroscopy and angiography provide dynamic information regarding fragment mobility and location. Management strategies depend on fragment position, mobility, associated complications, and procedural risk. Endovascular retrieval may be attempted if the fragment is accessible and not embedded.^1^ Surgical extraction is indicated in cases of chamber perforation, increased arrhythmia risk, or when the patient is already undergoing cardiac surgery, as occurred here.^2,3^ Observation may be considered only in carefully selected asymptomatic patients. In this patient, surgical removal was performed during the same operative session as coronary artery bypass grafting, while the remaining filter, previously deemed difficult to retrieve, was left *in situ* with planned longitudinal radiographic surveillance.

This case highlights persistent limitations in IVC filter follow-up and retrieval practices. The Society of Interventional Radiology (SIR) guidelines emphasize prompt retrieval of retrievable filters once protection from pulmonary embolism is no longer necessary, because prolonged dwell time significantly increases the risk of fracture and migration.^6,7^ Structured recall systems and dedicated follow-up pathways improve retrieval rates and reduce long-term complications. Earlier retrieval of this patient’s filter, which was placed during a period of high bleeding risk related to active cancer therapy, may have reduced the likelihood of this late presentation.

Ultimately, this case demonstrates that IVC filter complications can remain clinically silent for many years. It reinforces the importance of structured surveillance and timely retrieval and highlights the need for heightened awareness during invasive procedures where incidental findings may reveal otherwise undetected pathology.

## Patient perspective

The patient expressed relief that the fragment was discovered and removed before causing clinical deterioration and emphasized the importance of improved long-term follow-up for implanted devices.

## Lead author biography


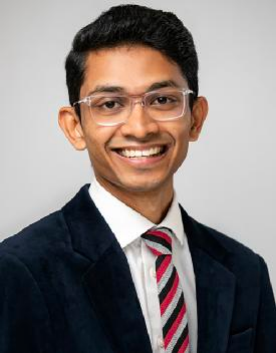


Benjamin M. Easow is an internal medicine resident at Southeast Health in Dothan, Alabama, with a strong interest in cardiology, particularly interventional and structural heart disease. He has authored over 30 abstracts and presented at various national and regional medical conferences. He is actively involved in clinical research, case reporting, and medical education. He is passionate about improving healthcare delivery and patient outcomes through evidence-based practice. Committed to pursuing a cardiology fellowship, he continues to contribute to academic medicine through scholarly activity and collaborative initiatives.

## Supplementary Material

ytaf657_Supplementary_Data

## Data Availability

No new data were generated. All information is included within the article and is available from the authors on reasonable request.
